# Medicine as a Study

**Published:** 1897-09-04

**Authors:** 


Vol. XXII.—No. 571.] [Sept. 4, 1897.

### Medicine as a Study.

The circumstances which determine a young man's choice of his future profession are almost infinitely various, and range over the whole fields of opportunity, of fitness, and of inclination. The latter may not infrequently be directed towards medicine by observation of the important part which is played by the doctor in many households, and by recognition of the manner in which his presence removes anxiety, or in which his skill assuages pain. "There are men and classes of men," wrote Robert Louis Stevenson, in his dedication of "Underwoods," "that stand above the common herds; the soldier, the sailor, and the shepherd not unfrequently; the artist rarely; rarelier still, the clergyman; the physician almost as a rule. He is the flower (such as it is) of our civilisation; and when that stage of man is done with, and only remembered to be marvelled at in history, he will be thought to have shared as little as any in the defects of the period, and most notably exhibited the virtues of the race. Generosity he has, such as is possible to those who practise an art, never to those who drive a trade; discretion, tested by a hundred secrets; tact, tried in a thousand embarrassments; and, what are more important, Herculean cheerfulness and courage. So it is that he brings air and cheer into the sick room, and often enough, though not so often as he wishes, brings healing."

In the presence of such appreciation as this, and it is felt, and in their own language uttered, by hun. dreds who have no power of finished expression, it is natural that the impulses of a generous boy should impel him towards a life in which he, too, may come to be looked upon with like regard, and to be expected with like eagerness. Few boys, again, few at least of those who are on the way to become Englishmen, could read such a book as "Two Years Ago" without a wish to emulate the exploits of its hero; or, turning from fiction to fact, which is stranger than fiction, could be unmoved by the history of Assistant-Surgeon Thompson, who remained, with only his soldier-servant, John McGrath, as a companion, for three days and nights upon the battlefield of the Alma, endeavouring to relieve the sufferings of the wounded; by that of Dr. Hayes, who took no small part in the desperate struggle at Rorke's Drift; by that of Landor at Majuba Hill, when, himself severely wounded, he yet found strength to place his skill at the disposal of others; or by that of Surgeon-Captain Whitchurch, whose bravery made him conspicuous among brave men in the heroic defence of Chitral. In an empire on which the sun never sets there is no place and no day in which the members of the medical profession may not be found at the post of duty, wherever this may be—in the palaces of the wealthy, in the slums of cities, in the lonely hamlet, in the beleaguered garrison, in the tented field. No other calling permits such intimate study of the essentials, as distinguished from the accidentals, of human nature; no other is calculated so to entwine the love of truth with every fibre of the intellectual organism; no other affords even approximately equal opportunities of doing good.

These are weighty considerations; but they must not be permitted to divert attention from the fact that the highest awards of medicine come only to those who will seek them with due competence and diligence, as well as by the appointed road. Macaulay, in his description of the bravery of William III., says that sufficient courage to go through a campaign without discredit is possessed, or with proper training may be acquired, by the great majority of men. It is, likewise, probably true that the great majority of men could be taught to fulfil, without discredit, the daily duties of a medical practitioner. It is certainly true that high professional distinction cannot be achieved (we do not speak of mere popularity or even of mere gain) except by the absolute devotion of mental faculties of a high order to the comprehension of the problems which medicine offers to its votaries. The life of a medical student must be one of assiduous application, not directed merely to success at examinations, but to the study of the changes wrought by disease in the human organism. A competent examiner has seldom any difficulty in distinguishing the candidate who has worked in order to know from the candidate who has worked in order to pass; but the daily self-examination of the student, the answer which his own conscience must give with regard to the character of his efforts, is usually of far greater importance to his career than the impression which those efforts may produce upon others. Idleness and inattention cannot be limited in their effects to himself and his own prospects, because they constitute dishonesty in relation to other people. There may be callings in which the idle or incompetent man is nobody's enemy but his own, but medicine is not one of them. The medical student seeks to obtain from the State a right to receive money in return for certain definite services; and if he fails by any fault of his own to qualify himself to render those services in an effectual and proper manner he is, so far, a cheat. Subject to this consideration, an important element in the suitability of medicine as a profession is the great variety of work which it affords, and the opportunities given by this variety to persons of different tastes and inclinations. The studious man will seldom find it impossible to attach himself to some medical school at which his leanings may at once be utilised and gratified; the man of accomplishments and *savoir faire* will find his opportunities among the more wealthy circles of great towns; the man of rural tastes may indulge them in country practice; the more adventurous spirits may find appropriate spheres of action in the army or navy, or in the new fields of discovery which are perpetually being thrown open by English enterprise. There need seldom be a question, in the profession of medicine, of putting a square peg into a round hole. The holes are not only numerous, but they are of all shapes and sizes, so that the young doctor who cannot find one to fit him must be either exceptionally angular or exceptionally unyielding. All alike, however, demand from their occupants one essential condition: the condition of earnest endeavour to become qualified in fact, and not only in name, for the fulfilment of such duties as the position finally accepted may present.

